# Application of viral vectors to the study of neural connectivities and neural circuits in the marmoset brain

**DOI:** 10.1002/dneu.22459

**Published:** 2016-10-26

**Authors:** Akiya Watakabe, Osamu Sadakane, Katsusuke Hata, Masanari Ohtsuka, Masafumi Takaji, Tetsuo Yamamori

**Affiliations:** ^1^ Laboratory for Molecular Analysis of Higher Brain FunctionBrain Science Institute, RIKEN2‐1 HirosawaWakoSaitama351‐0198Japan

**Keywords:** marmoset, virus vectors, neocortex

## Abstract

It is important to study the neural connectivities and functions in primates. For this purpose, it is critical to be able to transfer genes to certain neurons in the primate brain so that we can image the neuronal signals and analyze the function of the transferred gene. Toward this end, our team has been developing gene transfer systems using viral vectors. In this review, we summarize our current achievements as follows. 1) We compared the features of gene transfer using five different AAV serotypes in combination with three different promoters, namely, CMV, mouse CaMKII (CaMKII), and human synapsin 1 (hSyn1), in the marmoset cortex with those in the mouse and macaque cortices. 2) We used target‐specific double‐infection techniques in combination with TET‐ON and TET‐OFF using lentiviral retrograde vectors for enhanced visualization of neural connections. 3) We used an AAV‐mediated gene transfer method to study the transcriptional control for amplifying fluorescent signals using the TET/TRE system in the primate neocortex. We also established systems for shRNA mediated gene targeting in a neocortical region where a gene is significantly expressed and for expressing the gene using the CMV promoter for an unexpressed neocortical area in the primate cortex using AAV vectors to understand the regulation of downstream genes. Our findings have demonstrated the feasibility of using viral vector mediated gene transfer systems for the study of primate cortical circuits using the marmoset as an animal model. © 2016 Wiley Periodicals, Inc. Develop Neurobiol 77: 354–372, 2017

## INTRODUCTION

The neocortex has emerged specifically in mammals during the course of the evolution of their brain. The neocortex has six layers common to all mammals. In this regard, it shares features common to the Mammalian class. However, it also shows marked diversity. Among mammalian brain structures, the variation in size is most prominent in the neocortex. The size of the mammalian brain varies from the Etruscan Shrew (the smallest nonflying mammal) to the killer whale on the order of 10^4^‐fold difference. The brain size is approximately proportional to the surface area of the neocortex with the same order of difference (Stephan et al., 1986; Jerison, 2001). The mammalian brain consists of parcellations, so called areas, each of which reflects a specific unit of neuronal projections (Brodmann, 1909). Areas are formed by different intermediate levels of structures called columns, modules and domains, which are different among the different regionalization of the neocortex (Kaas, 2012). For example, not only increase in the size and volume of the prefrontal cortex but also the rich connections with other brain regions underlie the richness of human cognition (Fuster, 2015). The neocortex is the most evolved structure in primates compared with other animals (Brodmann, 1909), in a different way from ancestral mammals (Kaas, 2013). To understand the underlying mechanisms of brain functions, it is critically important to understand the connections and functions underlying neuronal activities and behavioral responses. Traditionally, this set of studies has been conducted in terms of neuroanatomy and neurophysiology. Recently, multidisciplinary approaches that include molecular biology, biochemistry, cell biology, and genetic engineering have been integrated to neuroscience with the traditional anatomical and physiological approaches (Kandel et al., 2012). We are interested in developing gene manipulation methods that can be used for the primate brain. Since biological materials such as DNA, RNA, and proteins are common properties among species, in principle the techniques that are applied to one species can also be applied to other species. Given that certain cortical structures and functions of the primate brain differ from those in other mammals (Nieuwenhuys, 1994; Fuster, 2015), it is important to develop techniques to study the primate neocortex. The pursuit of developing such techniques has been hampered because the reproduction system is different between mice and primates and, even though it becomes recently possible to generate transgenic and knockout primates (Sasaki et al., 2009; Niu et al., 2014), the number of genetically engineered animal models is still limited.

The recent availability of genetically engineered primates means that methods involving viral vector mediated gene transfer have increasingly become important because they can be used in combination with other methods using genetically engineered transgene lines, which mutually enhances markedly their advantages (Nassi et al., 2015a). Over the last several years, our laboratory has focused on developing viral vector systems for gene transfer. Because of their efficacy and low toxicity, we considered that Adeno‐associated virus (AAV) vectors is the best among the possible choices available (Nassi et al., 2015b; El‐Shamayleh et al., 2016). In addition, retrograde projection targeting has several specific purposes (EL‐Shamayleh et al., 2016). Since LV vector mediated retrograde transduction is more efficient than AAV vector mediated transduction in macaques, we used lentiviral (LV) vectors containing fusions of the extracellular domain of the rabies glycoprotein and the intracellular domain of the VSV glycoprotein for retrograde labeling (Kato et al., 2013).

Here, we review our recent approaches using viral vector mediated gene transfer systems for the primate brain. Merits of using marmosets in various aspects of research interest have been published (Miller et al., 2016; Homman‐Ludiye and Bourne, in press; Oikonomidis et al., in press; Hagan et al., in press). In this review we focus on the topics that are directly related to our recently reported applications of viral vectors for gene transfer in the primate cortex, particularly in the marmoset brain. There are excellent reviews that have been recently published and cover most topics that we will no longer review. We briefly introduce such reviews. Nassi et al. (2015b) report comprehensively reviewed most of the topics that are relevant to this article including the characteristics of the available viral vectors. AAV may be the most commonly used vector because it is useful for long‐term and high level expression in the CNS of mammals. Moreover, it is straightforward to engineer the virus. Therefore, we used only AAV as our choice for gene transfer purposes to neurons although there is a major limitation in the size of genes incorporated into AAV. To use AAV, there are several remaining issues that should be solved (Nashi et al., 2015b). Among them, the toxicity and cell‐type specificity of promoters are important issues, which we want to focus on in this review on the basis of our recent findings (Watakabe et al., 2014a). Note that there are many options depending on the situation that researchers face, as reviewed by Nashi et al. (2015b) and El‐Shamayleh et al. (2016). El‐Shamayleh et al. (2016) have recently reviewed the strategies for targeting neuronal circuits in primates. This is also very relevant to the topics that we review here in that they specifically review three topics on viral vector tropism, projecting targeting, and targeting neurons at the transcriptional level for the application of viral vector technologies in primates. Furthermore, Callaway and Luo (2015) reviewed more specific topics on monosynaptic circuit tracing with glycoprotein deleted rabies viruses that they have developed.

### Comparative Analysis of AAV Serotypes in Mice, Marmosets, and Macaques

**Figure 1:**
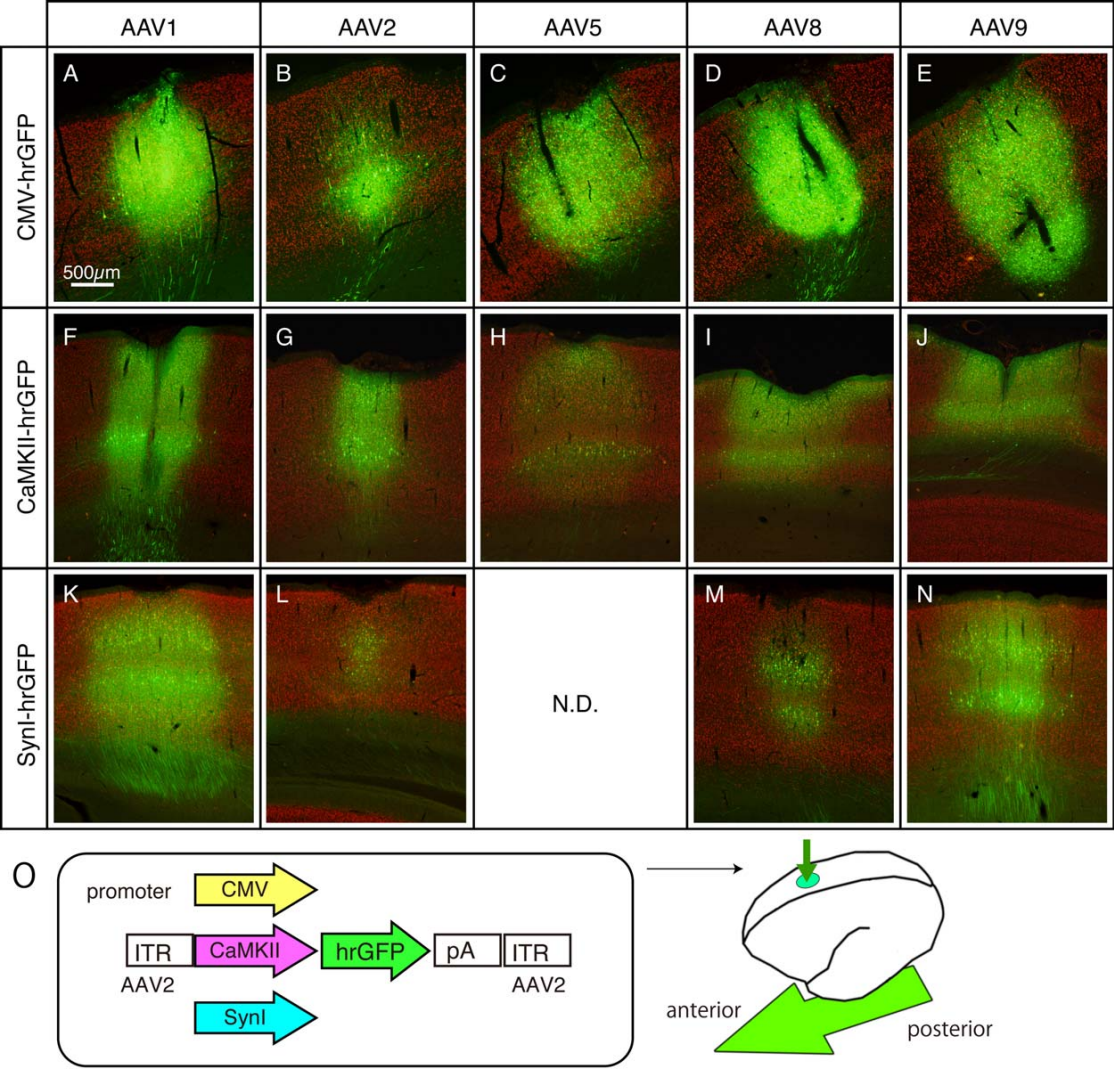
Various AAVs injected into marmoset cerebral cortex. AAV constructs containing CMV, CaMKII, and hSynI promoters driving hrGFP expression were packaged in AAV1, 2, 5, 8, and 9 capsids and injected into the marmoset cerebral cortex. The low‐magnified images of representative sections are shown for each combination. These are merged views for hrGFP (green) and NeuN (red) signals. To aid visualization, the contrasts of images are adjusted. So that we can directly compare the signal intensities across serotypes, the sections for the same promoters were immunostained simultaneously, imaged and contrast‐adjusted in exactly the same conditions. These images were taken from two marmosets of ID#1 and ID#2 for CMV promoter (panels A–E), ID#5 for CaMKII promoter (panels F–J) and ID#4 for hSynI promoter (panels K–N). Panel O shows the schematic views of the AAV constructs and injections. The arrow indicates the anterior–posterior axis. (For interpretation of the references to color in this figure legend, the reader is referred to the web version of the article sited <www.elsevier.com/locate/neures) (From Figure 1 of Watakabe et al., Neurosci. Res., 2014a, 93, 144–157). [Color figure can be viewed at wileyonlinelibrary.com]

As reviewed by Nassi et al. (2015b) and EI‐Shamayleh et al. (2016), AAV is the most common choice among the viral vectors available. However, there are several issues that remain to be solved in using AAV vectors. They include AAV serotypes, promoters, toxicity, and species specificity (Nassi et al., 2015b; EI‐Shamayleh et al., 2016). Nathanson et al. (2009) used an AAV serotype 1 capsid with a serotype 2 ITR backbone packaged serotype (AAV2/1) and lentivirus pseudotyped with the vesicular stomatitis virus (VSV‐G‐LV). They used human synapsin (hSyn) and a mouse CAMKII (CAMKII) promoter. A serial dilution of AAV1/2‐hSyn injected into the mouse somatosensory cortex (S1) shows different ratios of excitatory and inhibitory neurons that are infected. Higher infected titers of AAV1/2‐hSyn tend to infect more excitatory cells whereas lower infected titers of AAV1/2‐hSyn injections show higher rates of inhibitory neuronal infection. The same titers of AAV1/2CAMKII and VSV‐G‐LV‐hSyn yield high ratios of excitatory neuronal infection, whereas similar titers of AAV‐CAG injected yield a high ratio of inhibitory neuronal infection. They also found some neurotoxicity of VSV‐G‐LV‐hSyn. These findings demonstrated that many factors could affect the features of infection. We considered that it is important to determine the factors that influence the efficacies of gene transfer and tropism. To this end, we compared the infectious properties of five different capsids of AAV serotypes 1, 2, 5, 8, and 9 in marmosets as well as in mice and macaques. Initially, we used hrGFP under the control of the CMV promoter with AAV2 ITRs as the construct for capsid comparison (Cearley and Wolf, 2006; Taymans et al., 2007; Nassi et al., 2015b). We used neuron‐specific CaMKII, and hSyn1 promoters followed by hrGFP (Fig. 1). Each of the five serotypes showed differences in viral spread and cell tropism. Among the five serotypes, AAV2 showed the least spread of GFP; after the injection of 2.5 × 10e9 vector genome copies of AAV in 0.5µl PBS over 5 min, we observed an AAV2 spread of approximately 0.7 mm, whereas the spread of other serotypes was 1.2–1.3 mm. We observed no statistically significant differences in viral spread among AAV serotypes of 1, 5, 8 and 9. Regarding cell tropism, AAV2 exhibited a significant difference in neuronal tropism even with the CMV promoter in the three species tested. Other serotypes exhibited more or less glial expressions that obscured neuronal expression. For this reason, we employed neuron‐specific CaMKII and hSyn1 promoters to compare neuronal transduction efficiency among serotypes. We estimated the efficacy of infection of neurons by comparing between hrGFP‐ and NeuN‐positive cells. The efficacy of infection by injection with the CaMKII promoter was over 70% in all the serotypes. Thus, at least under our conditions, we observe no significant differences among AAV1, 5, 8, and 9, except that the neuronal transgene expression level was slightly lower in AAV5. We also observed no significant differences across species, although the infected glial cells exhibited different morphologies and may have species‐specific glial tropism.

Regarding the choice of promoters, we found strong toxic effects when AAV with CMV‐GFP constructs were injected. The toxicity was estimated by comparing the signal intensities of GFP (reporter), NeuN (neuron), and Hoechst33342 (nuclear staining) 3 and 6 weeks after the AAV injection. Cells infected CMV‐GFP showed a low signal intensity of GFP and almost no NeuN expression 6 weeks after infection and a high signal intensity of Hoechst33342, which suggest the degeneration of neurons and infiltration of reactive glia cells. No AAV toxicity was observed when the CaMKII and hSyn1 promoters were used. We, thus, recommend the use of these neuron‐specific promoters for long‐term experiments.

Another important aspect to consider when selecting promoters is the cell‐type specificity of transgene expression. Consistent with the previous reports by Nathanson et al. (2009) and Scheyltjens et al. (2015), we found that for both CaMKII‐and hSyn1‐GFP constructs, GFP was expressed in both parvalubumin‐negative and ‐positive cells, which are a major subclass of GABAergic neurons (Nathanson et al. 2009, Scheyltjens et al. 2015). For selective expression in excitatory neurons, a 0.4 kb version of the CaMKII promoter may be used (Scheyltjens et al. 2015; Gerits et al., 2015). It was conspicuous in the marmoset cortex that large pyramidal cells in layers 3 and 5 tend to strongly express the transgene, regardless of the promoter or serotype used. Expression of the CaMKII construct appears to be stronger than that of the Syn‐1 construct in small to medium pyramidal neurons.

There have been many studies on rodents and nonhuman primates in which different AAV serotypes have been tested (see Tables 1, 2, 3). The results of such studies were not always consistent. For example, we and others found glial expression of the CMV promoter for AAV8 and AAV9, whereas others reported neuronal tropism for these serotypes (Masamizu et al. 2011; Aschauer et al., 2013; Scheyltjens et al., 2015). We also found a good spread of AAV1 as opposed to a report showing a limited spread of this serotype (Scheyltjens et al. 2015). This discrepancy suggests a lack of understanding of the parameters that determine the physical and biological spread of AAV viral particles and how they transduce neuronal and non‐neuronal cells. Although we have successfully used AAV1 in marmosets (Sadakane et al., 2015a, 2015b), other serotypes can also work in combination with appropriate promoters, as our results demonstrated. AAV serotypes of 2, 5, 8, and 9 can be used in nonhuman primates (Diester et al., 2011; Cavanaugh et al. 2012; Jazayeri et al., 2012; Ohayon et al., 2013; Nassi et al., 2015a; Afraz et al., 2015; Inoue et al., 2015; MacDougall et al., in press; Matsuzaki et al., in press; Klein et al., 2016; Galvan et al., 2016). We expect that our studies would provide a basis for choosing an appropriate combination of a promoter and an AAV serotype for the marmoset brain depending on the experimental purpose. We have experience of using AAV vectors for other brain areas (e.g., thalamus and striatum), which gives some difference between different AAV serotypes. However, we have not systematically analyzed the serotype difference, which remains to be studied in future.

**Table 1:** Summary of AAV Serotpye Application in Mammalian Brains (mice)

<mice and rats>		AAV1	AAV2	AAV3	AAV4	AAV5	AAV6	AAV6.2	AAV7	AAV8	AAV9	AAVrh10	AAVDJ	AAVDJ8
cortex	CMV/CAG	1, 2, 3, 4, 9	1, 4, 9	9	9	1, 2, 4, 9	4, 9		2	1, 2, 4, 9	1, 2, 4			
	CaM(0.4/1.3)	1, 2, 3	1, 4			1, 2			2	1, 2	1, 2			
	Syn	1, 2	1, 4							1				
striatum	CMV/CAG	4, 11	4, 11			4, 11	4		11	4, 11	4			
	Syn									4				
hippocampus	CMV/CAG	4	4			4, 7	4		11	4, 7, 11	4, 7			
red nucleus	CMV/CAG	5	5	5	5	5	5			5				
substantia nigra	CMV/CAG	6, 8, 11	6, 8, 11			6, 8, 11		8	8, 11	6, 8, 11	8			
amygdala	CaM(0.4/1.3)	11	11			11			11	11	11	11	11	11

**Table 2:** Summary of AAV Serotype Application in Mammalian Brains (marmoset)

<marmosets>		AAV1	AAV2	AAV3	AAV4	AAV5	AAV6	AAV6.2	AAV7	AAV8	AAV9
cortex	CMV/CAG	1	1			1				1, 11, 12	1, 12
	CaM(0.4/1.3)	1	1			1				1	1
	Syn	1	1			13				1	1, 13
	mThy1S(TET)	20									
Striatum	CAG									11, 12	12
substantia nigra	CAG									11	
Cerebellum	Syn‐CMV, MSCV										22

**Table 3:** Summary of AAV Serotype Application in Mammalian Brains (macaques)

<macaques>		AAV1	AAV2	AAV3	AAV4	AAV5	AAV6	AAV6.2	AAV7	AAV8	AAV9
cortex	CMV/CAG	1, 30	1, 19			1, 28			17	1, 24	1
	CaM(0.4/1.3)	1, 17	1			1, 17, 23, 27, 28, 29			17	1, 17	1, 17
	Syn	26				15, 28					
	hThy1					15					
Striatum	CMV	14, 16, 18	14, 16	14	14	14, 16, 18	14			16, 18	
substantia nigra	CMV	14	14	14	14	14	14				
LGN						21					
superior colliculus	CAG									25	

Previous reports using AAV vectors in mice, marmosets, and macaques are listed. Numerical numbers in each box indicated the papers cited that are shown below the table. Cited papers are found in REFERENCES.

1:Watakabe et al. (2015), 2:Scheyltjens et al. (2015), 3:Nathanson et al. (2009), 4:Aschauer et al. (2013), 5:Blits et al. (2010), 6:McFarland et al. (2009), 7:Carty et al. (2010), 8:Van der Perren et al. (2011), 9: Hutson et al. (2012), 10: Holehonnur et al. (2014), 11: Masamizu et al. (2010), 12: Masamizu et al. (2011), 13: MacDougall et al. (2016), 14: Markakis et al. (2010), 15: Diester et al. (2011), 16: Sanchez et al. (2011), 17: Gerits et al. (2015), 18: Dodiya et al. (2010), 19: Inoue et al. (2015), 20: Sadakane et al. (2015), 21: Klein et al. (2016), 22: Matsuzaki et al. (2016), 23: Yazdan‐Shahmorad et al. (2016), 24: Afraz et al. (2015), 25: Cavanaugh et al. (2012), 26: Jazayeri et al. (2012), 27: Nassi et al. (2015), 28: Ohayon et al. (2013), 29: Ruiz et al. (2013), 30: Stettler et al. (2006).

### Tracing Neuronal Connections Using Retrograde Tracing Systems

**Figure 2:**
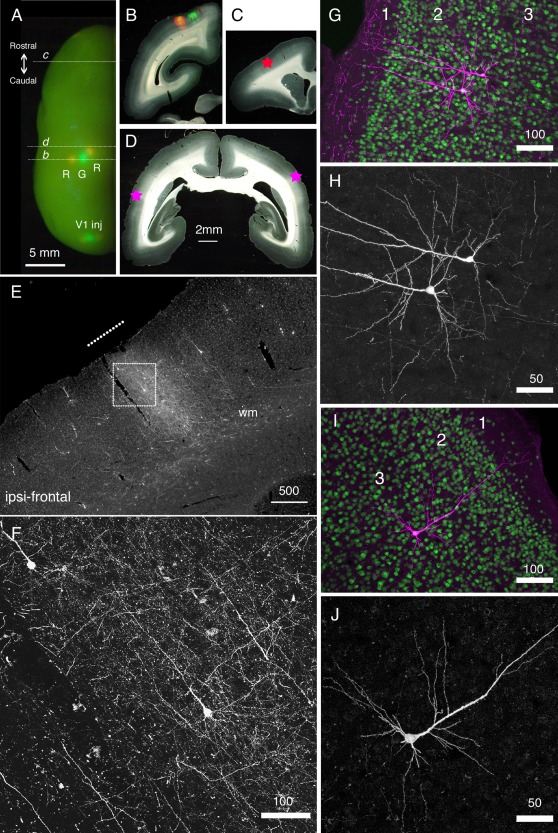
Long‐distance cortical connection of the marmoset brain visualized by StTTrR/FuG‐B vector. (A) The left hemisphere of the marmoset brain with cortical vector injections is shown from above. StTTrR/FuG‐B vector was injected into two sites in the parietal areas and two sites in V1 and StTTrG/FuG‐B vector was injected into one site in the parietal area and one site in V1, as indicated. The injection sites were visualized by LED illumination. (B–D) The coronal sections at the positions indicated in panel A. The fluorescent image is overlaid on the dark field image of the section in panel B. The asterisk in panel C shows the position of the image shown in panel E. The asterisk in the left side of the section in panel D corresponds to the ipsilateral side. (E) The frontal cortex ipsilateral to the vector injection. At this plane, the forward projection fibers of RFP were visible (shown by a white line in panel E), together with retrogradely labeled cell bodies. (F) Confocal image of the boxed region in panel E. Dense axonal fibers and dendrites were visible. This is the fluorescence image of RFP with no antibody enhancement. (G–J) Confocal images of the corticocortical neurons. RFP signals are enhanced by immunofluorescence (magenta) and counterstained with NeuN antibody (green) in panels G and I. Only the RFP signals are shown in panels H and J. The two neurons in panels G and H and one neuron in panels I and J are ipsilateral and contralateral to the injection site, respectively. Bar: 5 mm for A; 2 mm for B–D; 500 µm for E; 100 µm for F, G and I. 50 µm for H and J (From Figure 6 of Watakabe et al., PLOS ONE 7, e46157). [Color figure can be viewed at wileyonlinelibrary.com]

**Figure 3:**
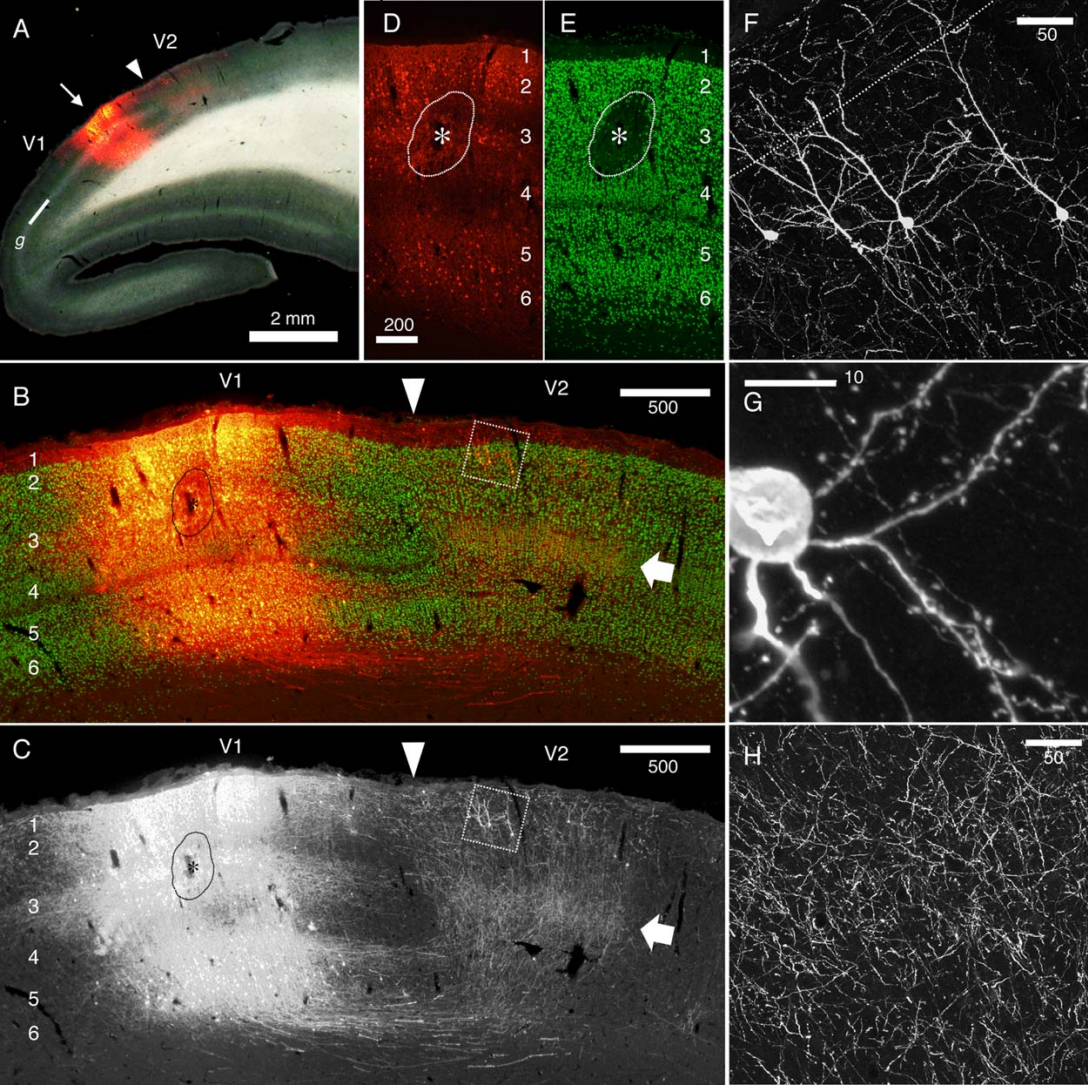
Reciprocal connectivity of marmoset V1 and V2 visualized by StTTrR/FuG‐B vector. The caudal block of the marmoset brain shown in Figure 7 was parasagittally sectioned to visualize the reciprocal connectivity between V1 and V2. (A) Dark field image overlaid with RFP fluorescence indicates the injection site. The V1/V2 border (shown by the arrowhead) was clearly delineated by the presence of the striate of Gennari (g). The arrow indicates the injection site. (B and C) The RFP signals around the injection site (red) are shown with NeuN conterstaining (green). The arrow indicates the plexus of V1 terminals in layer 4. The boxed region contains the retrogradely labeled V2 neurons with feedback projection to V1, which is magnified in panel F. The asterisk indicates the core of injection, where NeuN expression is lost by local damage. Only the RFP signals are shown in panel C. (D and E) RFP signals (red) and NeuN stains (green) in the injection center. The contrast for the RFP signals is adjusted so that the cell bodies of the infected neurons can be delineated. Note that the lamina positions of the infected cells are restricted mostly to layers 2, 3 and 6. (F–H) Confocal images of RFP in V2. RFP signals are enhanced by immunofluorescence. Panel F shows the cell bodies and dendrites of V2 neurons that project back to V1. Panel G shows the spines of the basal dendrites originating from one of these cells. Panel H shows the plexus of axon terminals concentrated in layer 4 of V2. Bar: 2 mm for A; 500 µm for B and C; 200 µm for D and E; 50 µm for F and H; 10 µm for G (From Figure 8 of Watakabe et al., PLOS ONE 7, e46157). [Color figure can be viewed at wileyonlinelibrary.com]

In an analogous way of Cre‐dependent tracing strategy (see Fig. 1 of Nassi et al., 2015b), we attempted to develop a system with which we can visualize cortical neuronal connections by amplifying the signals using the retrograde lentiviral vector system and TET‐OFF/TET‐ON system. This system consists of two components, namely, the tetracycline transactivator (tTA) under the control of cellular promoters, such as the neuron‐specific human synapsin 1 (hSyn1) promoter, and the tetracycline‐responsive‐element promoter (TRE) upstream of the transgene of interest. When these two transcriptional units are present in the same cell, the transgene expression is highly enhanced and becomes sensitive to tetracycline. We developed new lentiviral constructs that have two promoter units; the hSyn1 promoter regulating tTA and the TRE promoter regulating a GFP or RFP sequence separated by an insulator sequence (cHS4) (Watakabe et al., 2012). This system was packaged into a lentivirus with the fusion glycoprotein of the B type (FuG‐B) that contains a transmembrane domain of RV‐G and a cytoplasmic domain of VSV‐G (Kato et al., 2011a) for retrograde transport. We conducted a series of experiments on mice and applied this system to a marmoset. As shown in Figure 2, when this retrograde vector was injected into the left hemisphere of the marmoset brain, neuronal cell bodies were clearly identified in the ipsilateral and contralateral sides under a confocal microscope, which are considered the retrogradely infected neurons. When the same vector was injected into V1 of the marmoset (Fig. 3), fine subcellular structures of V2 neurons that send feedback projections to V1 were observed under a confocal microscope.

We also developed a new two‐vector system, with which neurons connecting two regions of interest can be selectively visualized (Watakabe et al., 2014b). For this purpose, two components of the TET‐Off system were separated into the retrograde lentiviral vector and AAV vector. The retrograde vector contained the MSCV promoter followed by tTA, which was pseudotyped with the fusion glycoprotein of the C type (FuG‐C) for neuron‐specific retrograde gene transfer (NeuRet) (Kato et al., 2011b). The AAV vector carried the TRE sequence followed by either TurboFP635(tRFP) or humanized recombinant (hr) GFP. When these vectors were injected into the thalamus and the cortex, we were able to specifically label corticothalamic neurons (Watakabe et al., 2014b). We also successfully applied the hSyn1h promoter followed by the TET‐ON reverse tetracyclin transactivator (rtTV16) as a local infection vector. In this case, TRE was followed by either synaptophysin (SYP) linked to cellulean (CFP) or turboFP635 (tRFP) as a retrograde vector with NeuRet. This system enabled the visualization of the extensive extrinsic and intrinsic axon collaterals of particular projection neuronal types comparably to fine Golgi staining. We used this system mostly in mice; however, may apply it to marmosets.

### Transcriptional Controls

Since the principle mechanism of genetic machinary is common throughout all organisms, genetic manipulation is a powerful general tool for studying the phenotypes of cells, neuronall circuits, and behaviors. There are many mechanisms that control gene expression, and these mechanisms can be categorized into DNA replication, RNA transcription, and protein translation for the inheritance and expression of genetic information from DNA (Watson et al., 2013). Among them, “targeting at the transcriptional level” is the most common strategy to control the expression of target genes. Therefore, a number of methods to control gene targeting using viral vectors have been developed and used in combination with genetic lines. Because the above‐mentioned reviews cover most of the current available techniques for this purpose (Callaway and Luo, 2015; Nassi et al., 2015b; El‐Shamayleh et al., 2016), we focus on two methods that we developed for applying to the primate brain: the use of the TET/TRE system to amplify two‐photon signals and the targeting RNA expression of a gene in the primate neocortex.

#### Application of TET and TRE System to Amplify Fluorescent Signals for Two‐Photon Imaging in Marmoset Cortex

**Figure 4:**
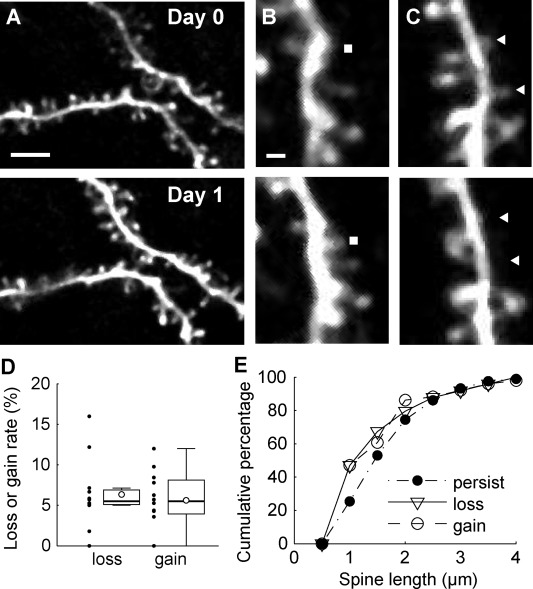
Time‐lapse imaging of spines in prefrontal cortex. A, The same dendritic regions in the prefrontal cortex were imaged at 24 h intervals. The top panel shows an image acquired on day 0 (7 d after craniotomy), and the bottom panel shows an image acquired on day 1. Scale bar, 5 µm. B, The gained spines were identified by manual inspection of two images acquired at 24 h intervals. A filled rectangle indicates the position of an example of a gained spine. Scale bar, 1 µm. C, The same as B for lost spines. Filled triangles indicate the positions of lost spines. D, Box plots showing the spine turnover rate. The open circles in box plots indicate mean values. Black dots indicate values for each site. The whiskers extend to the largest and smallest values within 1.5 times the interquartile range. E, Cumulative distributions of spine length in persisting, gained, and lost populations (From Figure 4 of Sadakane et al., eNeuro, 2015, 2(4), ENEURO.0019‐15).

Two‐photon imaging has an advantage in that long‐wavelength light can more deeply penetrate into a tissue specimen than short‐wavelength light, which is used in conventional microscopies such as confocal microscopy. Therefore, two‐photon microscopy have been applied to the study of systems of various organisms including mammalian nervous systems. For example, two‐photon microscopy was a key factor for the advancement of *in vivo* spine‐imaging (Denk et al.,^1990^; Denk and Svoboda, 1997). Two‐photon imaging has also been applied to primate brains. For example, there have been studies by two‐photon microscopy of the macaque cortex using virus expression systems (Stettler et al., 2006; Yamahachi et al., 2009). Although these studies revealed axonal structures, they did not show dendrite structures. We wanted to achieve dendrite imaging. For this purpose, we needed to establish two conditions. On one hand, dendritic signals should be strong enough to be observable. We have applied the TET/TRE system to amplify the fluorescence signals of the reporter with double infection, which is used originally in lentiviral vectors in mice (Hioki et al., 2009) of two AAV vector systems in the marmoset cortex. On the other hand, when positive signals are too crowded, dendritic signals are buried under strong cell and axonal signals. To overcome these apparently contradictory constraints, we applied the TET‐Off system carried by two AAV vectors for gene transfer. In this system, tTA was supplied by one AAV vector and TRE‐hrGFP was supplied by another AAV vector. These two vectors were combined at a certain ratio and injected to the marmoset cortex together. As mentioned above, TET‐Off system strongly enhances hrGFP signals. On the other hand, when we titrate the amount of tTA‐carrying AAV, we can make the transduction sparse, while maintaining the hrGFP expression level high. For this purpose, in collaboration with Dr. Kawasaki's group we used the Thy1S promoter to drive tTA expression, which is reported to sparsely label cortical neurons (Ako et al., 2011). We truncated a 5′ region of Thy1S by about 1.3 kb, which is reported to be nonessential (Vidal et al., 1990; Caroni, 1997), owing to the limited size of the inserted AAV vectors. As far as we tested, however, the Thy1S promoter was active in various types of cortical projection neuron including corticocortical, corticothalamic, corticopontine, and corticocollicular neurons in layers 2–6 as well as in inhibitory neurons (Sadakane et al., 2015a; AW, unpublished data). The ratio of Thy1s‐tTA to TRE‐hrGFP was changed from 1/200 to 1/2000. We chose 1/500 because the resulting signals gave the best resolution. By this method, we were able to obtain clear images of dendrites and spines by two‐photon microscopy. For example, we were able to observe the same dendrite and spines 24 h after the first observation (Fig. 4).

We then applied the Thy1S‐tTA and TRE systems to observe signals for calcium imaging. There were only limited studies on calcium imaging using genetically engineered calcium indicators (GECIs) in primates. One pioneering work on GECI application to the macaque cortex provided images for months through a chronically implanted window (Heider et al., 2010). However, only several neurons were imaged simultaneously in the same window and it appeared not possible to monitor the same neurons over long periods of time. Synthetic calcium indicator dyes have also been used for detecting the complex structure of macaque V1 (Nauhaus et al., 2012; Ikezoe et al., 2013). However, these synthetic dyes cannot be used for long‐term recording of the activity of neuronal populations and examining fine subcellular structures such as dendritic spines.

**Figure 5:**
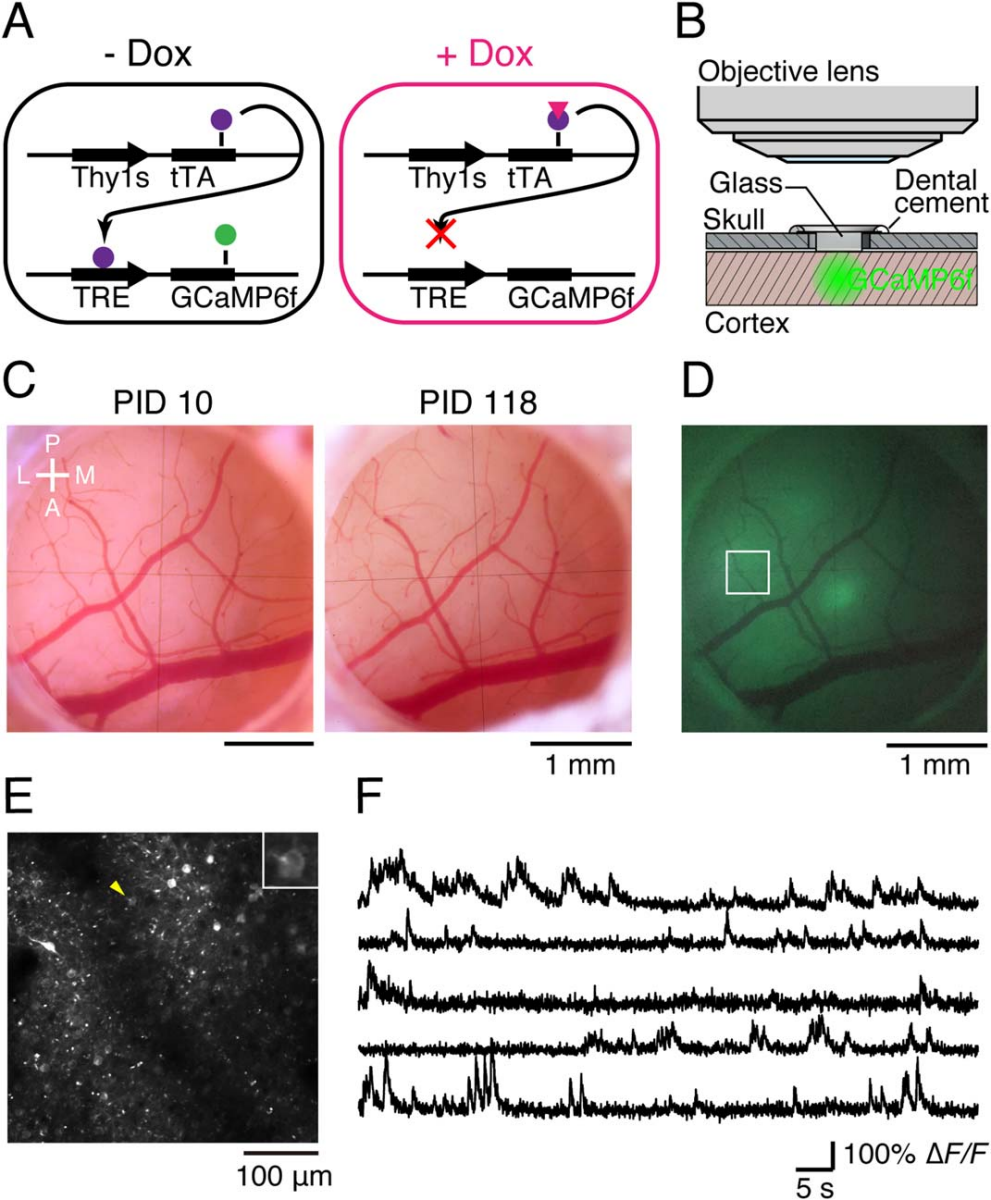
In vivo two‐photon calcium imaging in the marmoset neocortex using the TET‐Off System. (A) Schematic illustration of the TET‐Off gene expression system. tTA (purple circles) activates the TRE3 promoter to amplify GCaMP6f (green circle) expression. Dox (pink triangle) inhibits the binding of tTA to the TRE3 promoter. (B) Schematic illustration of the imaging window. “Glass” indicates four sheets of 3‐mm circular glass coverslips that were adhered to a 5.5‐mm circular glass coverslip. (C) Vasculature images from the same imaging window obtained on postinjection day (PID) 10 and PID 118. A, anterior; L, lateral; M, medial; P, posterior. (D) Epifluorescence image from the same window as shown in (C) on PID 10. The rectangle indicates the field shown in (E). (E) Two‐photon image of GCaMP6f in the rectangle shown in (D). The image was averaged across 4,500 continuous frames. The depth of the image was 250 μm from the cortical surface. The inset is an enlarged view of a single cell (yellow arrowhead). (F) Representative Δ*F*/*F* traces from five ROIs in the field shown in (E). The top trace is from the cell shown in the enlarged view in (E) (From Figure 1 of Sadakane et al., Cell Reports, 2015, 13, 1989–1999). [Color figure can be viewed at wileyonlinelibrary.com]

We applied the Thy1‐tTA and TRE‐GECI systems by double AAV infections in collaboration with Dr. Matsuzaki's group (Sadakane et al., 2015b; Fig. 5). We used GCaMP6f as a GECI because of its rapid response. Although an AAV vector that contains GCaMP6f driven by the hSyn1 promoter has been successfully used in mouse studies, the same viral preparation that worked in mice failed to exhibit detectable signal in the marmoset brain, even when injected at a very high dose. The application of a TET‐Off amplification system overcame this problem and we were able to observe strong signals of GECI in the marmoset cortex *in vivo*. Furthermore, these strong signals were controllable with doxycycline (Dox), a derivative of tetracycline. After applying Dox for 5 days (Marmoset A) and 7 days (Marmoset B), a marked decrease of fluorescence signals was observed in 10 and 15 days, respectively. Controlling the expression level is thought to be important to prevent the adverse effects of GECIs on expression (Tian et al., 2009; Chen et al., 2013). Indeed, we were still able to observe both GCaMP6f and NeuN signals 100 days after AAV injection, which confirmed that the expression of these markers in the same cells can be observed for a long period. We also used the TET‐ON system that carried hSyn1‐rtTA and TRE3‐GCaMpf. Without Dox administration, we observed no fluorescence signals, but 3 days after Dox administration we clearly detected epifluorescence. The intensity of signals returned to baseline levels within 2 to 3 weeks after the removal of Dox. We monitored neuronal activities in a broad area (625 × 625 μm^2^) at depths of 150, 275, and 400 μm. In an experiment, we identified 445 putative neuronal somata, 81 of which were spontaneously active (18/146, 29/168, and 34/131 at depths of 150, 275, and 400 μm, respectively).

**Figure 6:**
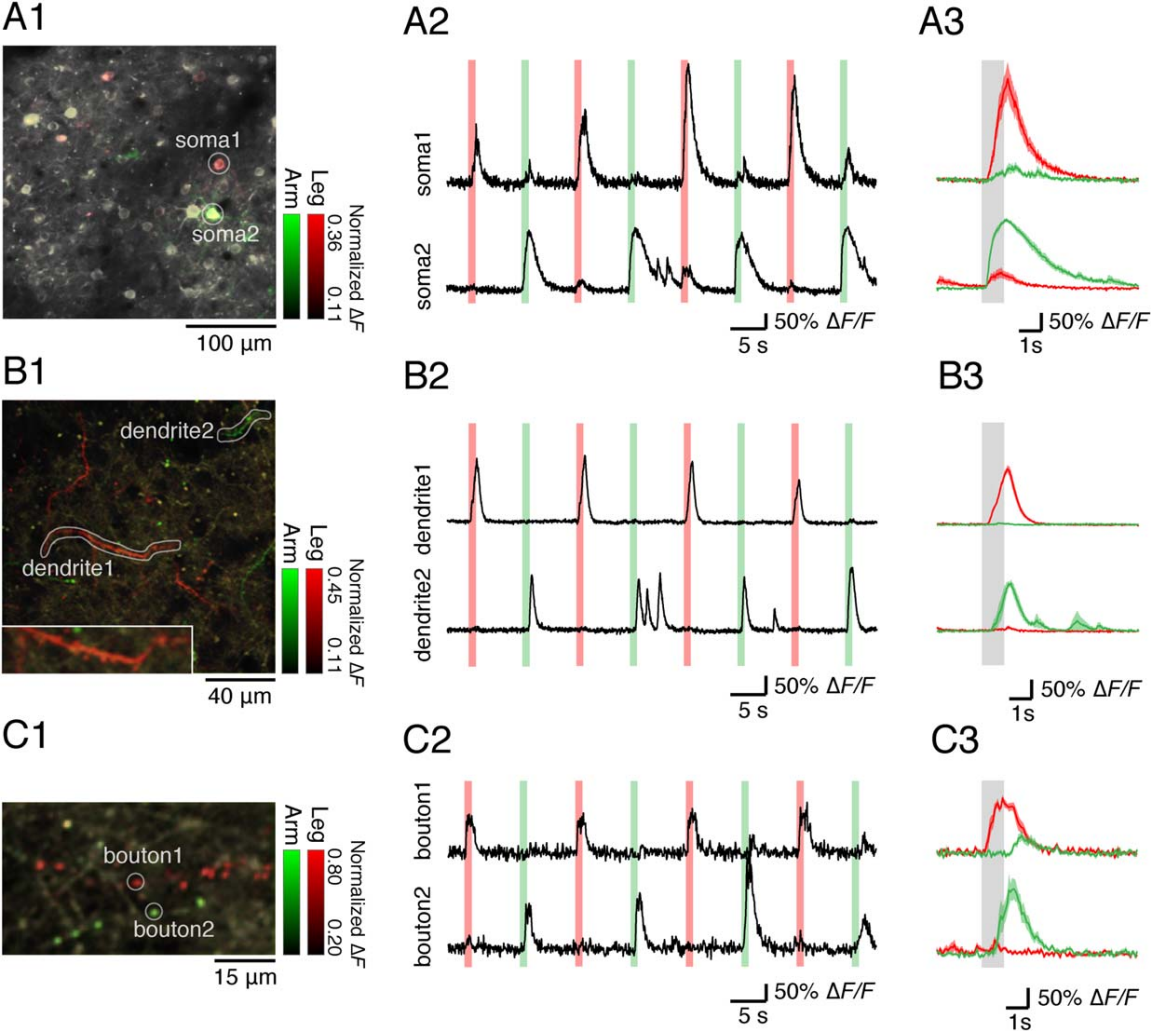
Selective Sensory Responses to Tactile Stimulation from Neuronal Somata, Dendrites, and Axonal Boutons. (A) Sensory responses to tactile stimulation from soma regions in the right somatosensory cortex. The depth of the image is 210 μm. (A1) Representative two‐photon images on which colored stimulus selectivity (green, left arm; red, left leg) is overlaid are shown. In each stimulation condition, the stimulus selectivity for each pixel was defined by dividing Δ*F* during the stimulation averaged across all trials by the maximum value of the averaged Δ*F* of all pixels in each field in both conditions. (A2) Representative Δ*F*/*F* traces from two neurons indicated in A1 (soma 1 and soma 2) are shown. The green and red lines indicate the timing of tactile stimulation to the left arm and the left leg, respectively. (A3) Averaged Δ*F*/*F* traces from soma 1 and soma 2 aligned to the left arm (green) and the left leg (red) stimulation are shown. The responses to nine stimuli to each body part were averaged. The gray band indicates the timing of the left arm and the left leg stimulation. (B) Sensory responses to tactile stimulation from dendrites in the right somatosensory cortex. The depth of the image is 15 μm. (B1) Representative two‐photon images on which colored stimulus selectivity (green, left arm; red, left leg) is overlaid are shown. (B2) Representative Δ*F*/*F* traces from two dendrites indicated in (B1) (dendrite 1 and dendrite 2) are shown. (B3) Averaged Δ*F*/*F* traces from dendrite 1 and dendrite 2 aligned to the left arm (green) and the left leg (red) stimulation are shown. (C) Sensory responses to tactile stimulation from axonal boutons in the right somatosensory cortex. The depth of the image is 20 μm. (C1) Representative two‐photon images on which colored stimulus selectivity (green, left arm; red, left leg) is overlaid are shown. (C2) Representative Δ*F*/*F* traces from two axonal boutons indicated in C1 (bouton 1 and bouton 2) are shown. (C3) Averaged Δ*F*/*F* traces from bouton 1 and bouton 2 aligned Cell Reports to the left arm (green) and the left leg (red) stimulation are shown (From Figure 6 of Sadakane et al., 2015, 13, 1989–1999). [Color figure can be viewed at wileyonlinelibrary.com]

We also detected neuronal activities in the somatosensory cortex evoked by tactile stimulation, attaching vibrators to the arm and leg of marmoset B contralateral to the hemisphere of the imaging window. We identified signals from soma, dendrites, and axonal boutons of neurons which selectively responded upon the stimulation of the leg and arm (Fig. 6). Our results proved that two‐photon microscopic technology is applicable to long‐term studies of neuronal circuits in the marmoset cortex.

There have been reports that applied two‐photon imaging using GECIs to animal species of larger brains than the mouse brain, including the rat (Scott et al., 2013), tree shrews (Lee et al., 2016), and ferrets (Smith et al., 2015). These studies used Synapsin promoter to drive the expression of GECIs similarly to the case of mouse, but in our hands, it was not possible to observe strong signals for two photon images in the marmoset cortex. We consider that this is because the marmoset brain is more opaque. It seems that at least three parameters, promoter activity, sensitivity of the calcium indicator, and the resolution of two‐photon microscopy are critical to obtaining good images and may be different between tissues. The best two‐photon images can be obtained improving each parameter and determining the optional combination of these parameters.

Optogenetics increasingly becomes an important technology used in the study of neural circuits (Fenno et al., 2011). The important issue related to two‐photon imaging is the optogenetical application to the primate cortex using viral vectors. Because these topics on the primate brain is well summarized in the review written by El‐Shamayleh et al. (2016), we will not repeat these in this review. However, it is worth noting that neuronal activities can now be successfully obtained by optogenetical techniques in awake marmosets using AAV vectors (MacDougall et al., in press).

#### Controlling Expression of a Specific Gene in a Region Specific Manner in the Primate Brain

Genetic knock out by homologous recombination is a powerful and standard method of altering the genome by homologous recombination (Capecchi, 1989), using conditional knock out technology (Lakso et al., 1992; Gu et al., 1994). Large scale resources are now available for obtaining appropriate target genes for conditional knock mice with pipelines (Skarnes et al., 2011). However, the situation is quite different for primates. Even though genetically engineered primate lines are generated, we have not reached the stage where we can knock out a particular gene in a region specific manner in primates. Therefore, we aim to establish a general method to manipulate any targeted genes. We therefore tested the methods to manipulate genes using gain‐of‐function and loss‐of‐function strategies. For this purpose, we chose a gene that controls other genes with high expression levels in representative brain areas. These area‐selectively expressed genes were identified by large‐scale screenings using differential display (DD) and restriction landmark cDNA scanning (RLCS) methods and classified into two groups: genes highly expressed in the primary visual cortex and those highly expressed in association areas (Yamamori, 2011). We first identified one of the V1‐selective genes as occipital 1 (*OCC1*), which was highly expressed in the primary visual cortex (Tochitani et al., 2001). Its function had long been unclear until a study of its role in the rodent dorsal root ganglion (DRG) revealed that follistatin‐like 1 (*FSTL1*) (mouse homolog of *OCC1*) is a Na‐K‐ATPase and controls afferent‐dependent synaptic transmission of sensory inputs to motor neurons (Li et al., 2011). Note that the expression of *OCC1/FSTL1* in the primate visual cortex is activity‐dependent, whereas none of the examined brain areas show activity dependency of *OCC1/FSTL1* (Takahata et al., 2006, 2008).

Although the molecular features of genes of each group show no apparent correlation, their expression patterns are very similar. This observation raised the possibility that there may be a common mechanism that controls the expression of these genes. To confirm this possibility, we examined the methylation of CpG islands in the promoter region of the area‐selective genes (Hata et al., 2013). The methylation levels of CpG islands of the promoter of V1‐selective genes (*HTR1B, HTR2A*, and *OCC1/FSTL1* genes) are low (Tochitani et al., 2001; Watakabe et al., 2009) and those of association area‐selective genes *(RBP4*, *PNMA5*, and *SLIT1* genes) are high (Komatsu et al., 2005; Takaji et al., 2009; Sasaki et al., 2010). However, there was no significant difference in the methylation level of each gene among brain areas (prefrontal area and V1). If methylation indeed plays any role in controlling the expression of area‐selective genes, there should be a mediator. We examined the expression patterns of five methyl‐binding proteins (MBDs) in the macaque cortex. Among five MBDs, only *MBD4* showed area selectivity. That is, the *MBD4* expression level is high in the prefrontal cortex and low in V1. Chromatin immunoprecipitation assay (ChIP) of the selective binding of a particular protein to the promoter region by PCR amplification analysis using a specific pair of primers after precipitating the complex with a specific antibody revealed the specific binding of MBD4 to the promoter regions of *PNMA5, RBP4*, and *SLIT1*. To determine whether MBD4 plays a critical role in controlling the expression of association‐area‐selective genes, we used a human neuroblastoma cell line (SH‐SY5Y). In SH‐SY5Y cells, the *PNMA5* gene is highly methylated while *MBD4* is poorly expressed. Demethylation by 5‐azacytidine diminished the binding of MBDs including MBD4, while. *MBD4* overexpression enhanced MBD4 binding, as determined by ChIP assay, and increased *PNMA5* expression levels, as determined by PCR assay. These assays using cultured neuroblastoma cells revealed that MBD4 does indeed control *PNMA5* gene expression by binding to the methylated site of the *PNMA5* promoter.

We then examined whether MBD4 controls the association‐area‐selective genes in vivo on the basis of gain‐of‐function and loss‐of‐function. We employed an AAV1‐vector‐mediated gene transfer system. For the study of loss‐of‐function, which means that we specifically targeted a gene that is already abundantly expressed, we conducted the RNA interference method using a short hairpin (sh) RNA against the *MBD4* gene. Sh‐RNA is under the control of the U6 promoter (Toro Cabrera and Mueller, 2016). For the study of gain‐of‐function, we express a gene of *MBD4* in V1 where its expression level is low. Introducing *MBD4* shRNA to the frontal cortex, in which BMD4 is highly expressed, reduced the *PNAMA5* and *RBP4* expression levels, but *SLIT1* expression was not affected. In the core region of the injected site, the *MBD4* signals were enhanced. The *MBD4* signals at the injected site likely arose from proliferating glial cells, as shown by the expression of the glial cell marker AIF1/Iba1 coinciding with the *MBD4* signals. Introduction of *CMV*‐*MBD4* to V1, where the *MBD4* expression level is low, induced the overexpression of *MBD4*, which in turn induced the expressions of *PNMA5* and RBP4 but did not affect *SLIT1* expression. We concluded that MBD4 controls the association‐area‐selective gene expressions of the *PNMA5* and RBP4 genes but not that of the *SLIT1* gene. Although the *SLIT1* expression level is high in association areas, the expression of *SLIT1* in cortical layers is complimentary to those of *PNMA5* and *RBP4* (Sasaki et al., 2010). That is, the expression level of *SLIT1* is high in layer 4 whereas those of *PNMA5* and *RBP4* are low. We speculate that there may be other factors that control *SLIT1* differently from *PNMA5* and *RBP4*, although *SLIT1* is also highly methylated and expressed in an association‐area selective manner. Our approaches using an AAV vector successfully proved the control of a particular gene in the primate neocortex, which can be used for the regional control of a specific gene in the primates brain where transgenic lines are hard to be available.

### Problems to Be Overcome in Future Studies

We have reviewed our recent studies focusing on the application of viral vectors to the study of neuronal connections and gene functions in the marmoset cortex. Toward this goal, we have developed and modified several techniques so that we can use the viral vectors for the study of neuronal circuits in the marmoset brain, which includes the following methods that we described in this review.

1. For the application of the AAV vector to the marmoset cortex, we compared AAV serotypes in combination with one of three different promoters of CMV, CaMKII, and hSyn1 for the gene transfer experiments on the cortices of mice, marmosets, and macaque monkeys. Each combination gives different results; therefore, the best combination should be chosen depending on the purpose of the experiment.
2. For the visualization of neuronal connections, we have used target‐specific double infection techniques in combination with TET‐ON/TET‐OFF systems. This set of systems works well in mice. Although the application of these techniques to the marmoset brain are still limited, they can be used for visualizing the primate neuron connections when combined with infection using TVA‐expressing systems (Callaway and Luo, 2015).
3. We were able to observe the subcellular morphology and activity in the marmoset brain by two‐photon microscopy using the Thy1S‐tTA and Tre‐GFP/Tre‐GCaMP6f systems. Promoter activity, sensitivity of Ca indicator sensitivity, and two‐photon microscopy resolution are critically important factors and should be continuously improved in future studies.
4. We also established an AAV‐mediated gene transfer method as a tool for the study of gene regulation in the primate neocortex by targeting the expression of a particular gene using either a loss‐ or gain‐of‐function strategy. This is particularly important as models of germline‐transmitted gene manipulation become available in primates (Sasaki et al., 2009; Liu et al., 2016) because virus‐mediated systems should be developed together with transgenic and knockout lines, which have been demonstrated in several animal models including mice.

Our studies clearly demonstrated the feasibility and usefulness of viral vector‐mediated gene transfer and manipulation systems. We have used AAV vectors, which seem to be the most appropriate except for retrograde tracing. However, there are still some concerns with using AAV vectors, which need to be improved in future studies as follows.

1. The major concern is the size limitation for insertion in AAV vectors. The size of the gene to be inserted is limited to approximately 4.8 kb (Dong et al., 2010). Although the high titer of coinfection of the AAV vectors that carry different exons could produce a larger protein (Wu et al., 2010), an insert size of more than 4.8 kb markedly decreases transfection efficiency and cannot provide consistent transfection efficiencies in our hands. Therefore, if an insert larger than 4.8 kb is required, an adenovirus or a lentivirus would be a better choice. However, these viruses cause other problems of low transfection efficiency and toxicity compared to AAV. Therefore, there is no complete solution at this point for integrating a DNA larger than 4.8 kb. If this problem is solved, it will greatly increase the availability of viral vector mediated gene transfer.
2. The above problem that DNAs larger than 4.8 kb cannot be integrated also causes the related problem of promoter specificity. As in the case of the three different promoters of CMV, CAMKII, and hSyn, none of them showed a complete specificity for a cell‐type although each promoter tended to show variability in specificity. As discussed above, it is not easy to overcome this size limitation. However, there are a few examples of studies using shorter promoter regions that maintain at least reasonable specificity (Kawashima et al., 2014; Zhang et al., 2015). It would greatly help in future studies to collect such an array of promoter sequences that demonstrate high specificity. This line of efforts will be critically important given that the insert size limitation cannot be easily overcome.
3. AAV vectors are less toxic than other viral vectors (Nassi et al., 2015b; EL‐Shamayleh et al., 2016). This is the reason why we chose AAV vectors in a series of our studies. Nonetheless, they are toxic to a certain degree as we described above for long‐term AAV infection. Their toxicity is particularly significant when a promoter with high activity such as CMV is used. One compromise to this toxicity may be to use an AAV2 capsid, which seems to be less viral spread in the cortex compared to the other AAV serotypes. Therefore a certain balance between serotype, promoter activity, and toxicity, which should be carefully evaluated depending on the target and objectives of experiments in each species.

The authors thank all the collaborators cited in the text.
